# Findings from a Randomized Pilot of a Full Factorial Trial of ASCENT: Acceptability, Feasibility, and Intervention Adherence

**DOI:** 10.21203/rs.3.rs-9118090/v1

**Published:** 2026-05-13

**Authors:** Karly M. Ingram, Rachel Glock, Lane Williamson, Reid Anctil, AnneMarie Coffey, Savanah Tribbe, Holly Holder, Alexander M. Schoemann, Grace Westcott, Antonija Augustinovic, David Victorson, DerShung Yang, Darla Liles, John M. Salsman

**Affiliations:** East Carolina University; East Carolina University; East Carolina University; East Carolina University; East Carolina University; East Carolina University; East Carolina University; East Carolina University; Virginia Commonwealth University; East Carolina University; Northwestern University Feinberg School of Medicine; BrightOutcome, Inc; East Carolina University Brody School of Medicine; Wake Forest University School of Medicine

**Keywords:** Adolescent and Young Adult, Cancer Survivors, Digital Mental Health, Feasibility, Acceptability

## Abstract

**Background:**

Adolescent and Young Adult Cancer Survivors (AYACS) are at a heightened risk for depressive symptoms, yet tailored mental health interventions for this population remain scarce. To address this critical need, the AYA Survivors’ Coping and Emotional Needs Toolkit (ASCENT) was developed as a digitally delivered intervention explicitly designed to reduce depressive symptoms for AYACS. In preparation for a full factorial trial of ASCENT, we conducted an NCI-funded (R00248701) pilot feasibility study (NCT06420193) to evaluate the acceptability and feasibility of our study procedures.

**Methods:**

Sixteen cancer survivors aged 15–39 who had completed treatment within the last 5 years were recruited from a rural academic medical center to participate in a 6-week mixed-methods randomized pilot study of a full factorial trial. Acceptability, feasibility, and adherence to study procedures were evaluated via self-report questionnaires, interviews, and usage data. To proceed with the full factorial trial, we required that at least 68% of participants provide average rating ≥ 3 on an investigator-developed acceptability survey administered at follow-up. Further, we required a minimum recruitment rate of 15% and a minimum retention rate of 68% to indicate that the planned trial will be feasible.

**Results:**

Thirty-eight AYACS completed screening, 33 of whom were eligible and 16 of whom enrolled and were randomly assigned to 1 of 16 conditions (1 participant/condition). Participants were blinded to which condition they had been assigned. Study procedures demonstrated high acceptability that exceeded the a-priori benchmark, as 11/12 (91.67%) participants that completed the follow-up questionnaire had a mean acceptability score ≥ 3, exceeding the a priori benchmark of 68%. However, both qualitative and quantitative data highlighted dissatisfaction with the length of the weekly and follow-up questionnaires. Feasibility metrics (recruitment rate = 19%; retention rate = 75%) exceeded a-priori benchmarks.

**Conclusions:**

While recruitment and retention procedures were rated as acceptable and recruitment and retention rates indicated the full factorial trial is feasible, the larger trial would likely benefit from fine-tuning the existing recruitment and retention strategies. Qualitative insights also provided critical recommendations for improving recruitment and retention, such as the need for additional information pre-enrollment and reminders to complete study procedures throughout the trial. This study identified key considerations for the conduct of future complex trials with AYACS.

## 1. Introduction

### 1.1. Background

As of 2020, there were an estimated 2.1 million adolescent and young adult cancer survivors (AYACS), diagnosed between the ages of 15 and 39 years (1). The National Cancer Institute estimates that there will be approximately 85,000 newly diagnosed AYACS in the United States in 2025 (2). Although rates of cancer in AYACS are growing, cancer survival rates at large have been improving (3). For AYACS, the 5-year survival rate is 86% (2), and as a result the number of AYACS is growing. However, there is a disparity between the number of AYACS and the availability of tailored interventions to address the unique psychosocial needs of this population. Cancer during adolescence and young adulthood often disrupts key developmental milestones, such as growing one’s autonomy, pursuing higher education and/or a career, and starting a family (4). Disruption of these milestones may contribute to a greater risk for depression, which is more prevalent in AYACS than adolescents and young adults (AYAs) without cancer and older cancer survivors (5, 6). Of note, approximately 1 in 4 AYACS report experiencing symptoms of depression, up to five times more than individuals diagnosed with cancer later in life (7, 8).

Despite the increased risk for depression in AYACS, interventions specifically targeting depressive symptoms in this population are currently insufficient. Many investigations studying the psychosocial needs of cancer survivors have primarily focused on older breast cancer survivors (9), demonstrating a need for additional research evaluating efficacy in survivors of other types of cancer and AYACS at large. Additionally, psychosocial interventions for AYACS have typically been delivered during business hours, diminishing their accessibility due to other engagements (e.g., school, work) (10).

### 1.2. Digital Interventions

A limited quantity of online interventions for AYACS has been studied, however, most interventions do not specifically target depressive symptoms and instead focus on other psychological outcomes (e.g., positive emotion skills, mindful self-compassion, feelings of resilience) (11-16), highlighting the need for depression-specific digital interventions. Furthermore, online interventions do not always focus on survivorship, instead including both patients undergoing active treatment as well as survivors despite their differing contexts and needs (15, 17). To date, findings from only one digital intervention targeting depression have been published (17). This study recently demonstrated the feasibility and preliminary efficacy of technology-assisted CBT for reducing depression in a pilot randomized controlled trial of 17 AYACS (Hedges’ g = 1.12). Notably, while intervention content was delivered digitally, participants also had a human coach to reinforce the concepts of the CBT intervention, increase personalization, and ensure the safety of the participant. Overall, this study found that participants who received the technology-assisted CBT intervention had significantly lower depressive symptoms compared to participants in the control group (17).

While there is promise in digital mental health interventions, there remains a critical need for rigorous development and evaluation of tailored interventions targeting depression for this population. Most online interventions developed to address psychosocial needs in AYACS have only been tested in small studies focused on evaluating intervention feasibility and acceptability. Moreover, data collected to establish the feasibility of study procedures for two-arm randomized controlled trials is often limited to simple metrics (e.g., recruitment rate, retention rate). Few studies with AYACS have utilized more complex designs such as full factorial trials and existing trials often do not conduct feasibility and acceptability pilots on study procedures. As such, it is important to establish the feasibility and acceptability of study procedures before investing resources into the implementation of larger, more complex trials. This aligns with established frameworks for intervention development, including the multiphase optimization strategy (MOST) (18).

### 1.3. Present Study

The present study utilized the MOST framework in order to pilot the feasibility and acceptability of the study procedures of a full factorial trial employing a digital tool to target depression in AYACS. The MOST framework consists of three phases: 1) preparation, 2) optimization, and 3) evaluation. The preparation phase is focused on designing and pilot testing intervention components. This is followed by the optimization phase in which a factorial trial identifies the most effective components of the pilot. Lastly, the evaluation phase entails study staff testing the efficacy of the intervention (18). Guided by this framework and user-centered design, we developed the AYA Survivors’ Coping and Emotional Needs Toolkit (ASCENT). ASCENT includes adaptations of four evidence-based interventions for depression (behavioral action, cognitive restructuring, mindfulness training, and positive psychology strategies) into components for digital delivery to AYACS (19). Building on the feedback gained from the co-design workshops and individual interviews, our team examined the feasibility and acceptability of study procedures (19, 20). Further, we evaluated participant adherence to completion of assigned intervention content. This trial was registered on ClinicalTrials.gov (NCT06420193) and a detailed protocol manuscript was submitted for publication prior to completion of study procedures (21).

## 2. Materials & Methods

## 2.1. Study Overview and Design Rationale

The current study used a mixed-methods approach, which allowed us to gain insight into the acceptability of study procedures, the feasibility of study procedures (i.e., recruitment rate, retention rate), and adherence to the intervention. Sixteen participants were recruited from an academic medical center in the Southeastern United States, and all study procedures were conducted remotely. As previously described (21), the sample size of N = 16 was set primarily for practical reasons and not driven by hypothesis testing or allowing for precise effect size estimates, consistent with our goal of assessing the feasibility and acceptability of study procedures. Once recruited, participants were to one of 16 experimental conditions an order randomly generated by AMS (21). Consistent with the methodology of a full factorial trial, each condition included a different combination of the five intervention modules within ASCENT: Feeling, Doing, Thinking, Being, and Positivity. Sixteen participants were enrolled in two separate and subsequent waves (*n* = 8 per wave) to allow for the opportunity to correct any unexpected issues with study procedures. Wave 1 recruitment occurred from Recruitment for this randomized pilot took place from 8/30/2024 to 11/13/2024 (follow-up completed 1/7/2025), while wave 2 recruitment took place from 1/7/2025 to 5/5/2025 (follow-up completed 6/15/2025). Recruitment was discontinued upon reaching our target sample size of 16. All study procedures were reviewed and approved by the East Carolina University and Medical Center Institutional Review Board (UMCIRB#: 23-001795).

### 2.2 Intervention Components

**Figure 1:**
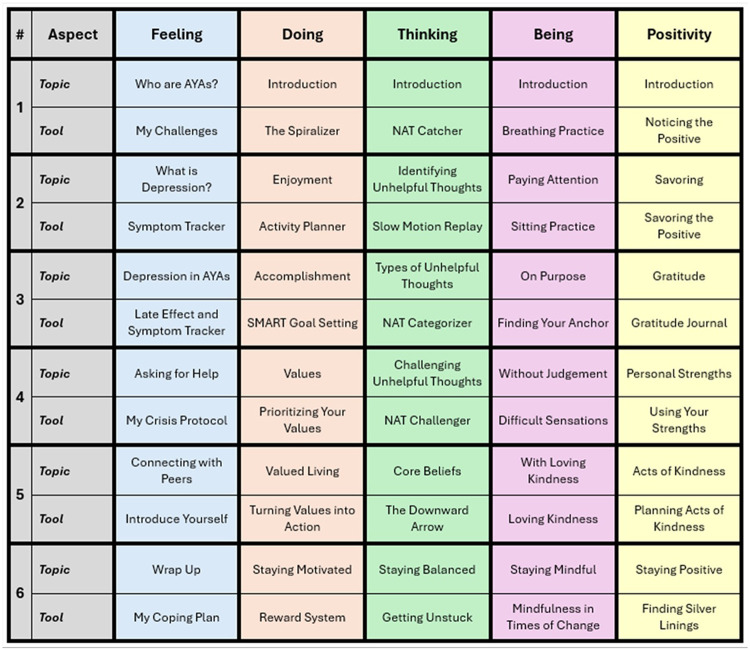
High-level summary of ASCENT content by component.

ASCENT includes a constant component that includes psychoeducation and the base features of the digital tool (called *Feeling*), as well as adaptations of four evidence-based interventions for depression (22): *Doing*, which was adapted from behavioral activation (23); *Thinking*, which was adapted from cognitive restructuring (24); *Being*, which was adapted from mindfulness-based interventions (25); and *Positivity*, which was adapted from a suite of positive psychology interventions (11, 26). Each intervention component consisted of six lessons; each lesson included (1) an animated video that provided educational information about the topic; (2) a story from a real AYACS that was related to the topic in the form of either a video or blog post; (3) multiple-choice questions that asked the participant to apply what they learned to the AYACS story; (4) open-ended reflection questions that asked the AYACS to consider how what they learned was relevant to them; and (5) a tool that could assist the AYACS in putting the information they learned into practice in their everyday life. ASCENT previously been described in greater detail (21); however, a high-level summary of intervention content is provided in Fig. 1. detail Participants had access to all assigned content via the ASCENT platform throughout the 6-week intervention period.

### 2.3. Procedures

#### 2.3.1. Eligibility Criteria

To be considered eligible for this study, individuals were required to 1) be between the ages of 15 and 39 years old at the time of screening, 2) have been diagnosed with cancer between the ages of 12 and 17 years old (adolescents aged 15–17) or 15 and 39 years old (emerging adults aged 18–25 and young adults aged 26–39), 3) be one month to five years post active cancer treatment at the time of screening, 4) be fluent in spoken and written English, and 5) own a smartphone with a data plan. Potential participants were excluded from the study if they were diagnosed with a severe or persistent mental illness or if they had suicidal ideation with a plan and intent at the time of screening.

#### 2.3.2. Patient Identification and Screening

Potentially eligible participants were identified by study staff via an Electronic Medical Record (EMR) review using International Classification of Diseases (ICD-10) codes for cancer (i.e., codes beginning with C) within the ECU Health Electronic Patient Information Center (Epic) database (27) and our local cancer registry. Trained study staff conducted chart reviews to assess eligibility based on confirmed cancer diagnoses, dates of diagnosis, and dates of last treatment. Physician approval was required before contacting potentially eligible individuals. Physician approval was considered obtained when the contacted provider responded with clear permission to contact the participant, or if the provider did not respond to the Epic message within three days of the message being sent. If a physician indicated that a patient was not appropriate for this study, the patient was not contacted.

#### 2.3.3. Recruitment

Methods for contacting patients included patient portal messages, emails, and phone calls. A systematic, multi-attempt protocol was employed with up to five contact attempts without response per individual, with the contact method escalating from electronic messages to phone calls. All recruitment and screening efforts were documented in the Research Electronic Data Capture (REDCap) database and a central progress spreadsheet. Once identified, potentially eligible participants over the age of 18 were contacted to complete an investigator-developed eligibility survey. For adolescent (aged 15–17) participants, parent/guardian permission was requested and received prior to first contact. Eligible participants were provided with an informed consent document through REDCap (28, 29). Participants over 18 years of age completed the consent form, while adolescents completed an assent form after parent/guardian permission was obtained.

#### 2.3.4. Orientation and Enrollment

Upon consent/assent, participants completed a baseline survey containing demographic, clinical, and medical history. After completing the baseline measure, a virtual orientation call was scheduled for the participant with a study staff member (KMI, RG, LW, RA, or AC) via Microsoft Teams (30). During this call, study staff reviewed the participant’s proposed assessment schedules, randomized the participant, created the participant’s account in ASCENT, introduced the ASCENT platform, provided an overview of the intervention components to which the participant was assigned based upon randomization, and set up the payment system for the participant. Study staff were instructed not to check the randomization scheme until the orientation call had been confirmed. Participants were considered enrolled in the study once they were randomized. As previously described (21) and in accordance with planned procedures for the full factorial trial, participants were randomized to one of 16 conditions and blinded regarding assignment to interventions. Participants were eligible to receive up to $150 total for their participation in the study ($30 for baseline completion, $5 for completion of each of 5 weekly surveys, a $15 bonus for completing all 5 weekly surveys, $40 for completion of the follow-up survey, and $40 for completion of the exit interview).

#### 2.3.5. Intervention and Weekly Assessments

Following the orientation call, participants were asked to use the intervention for six weeks, completing one lesson for each assigned component per week. Data related to adherence to study procedures was collected via the ASCENT platform and REDCap. Participants completed weekly measures of depressive symptoms and hypothesized mediators via weekly REDCap surveys during the intervention period. Specific measures used throughout the study have previously been described in detail (21).

#### 2.3.6. Follow-up Assessment & Exit Interview

At the end of week six, participants completed a comprehensive follow-up assessment and a semi-structured exit interview to gain insight into their experience with ASCENT. The acceptability measure was administered during the follow-up assessment. Interviews were recorded and auto transcribed using Microsoft Teams (30). Following data collection, study staff cleaned the generated transcripts by simultaneously listening to the audio and reviewing the text to ensure accuracy of transcriptions.

#### 2.3.7. Data Management and Security

All data was collected and managed electronically using HIPAA-compliant platforms (REDCap, Microsoft Teams, and ASCENT) (28-30). Data from these platforms were downloaded and stored on a secure university-managed drive only accessible onsite or via VPN. Auto-generated transcripts were cleaned and de-identified by study staff in preparation for qualitative analysis.

### 2.4. Data Collection

#### 2.4.1. Method

Data were collected via three methods: 1) REDCap, 2) semi-structured interviews, and 3) the ASCENT platform. Semi-structured interviews and select components of the REDCap survey were used to assess the acceptability of study procedures. REDCap survey completion was used to track recruitment and retention rates of study participants. Usage data from ASCENT provided information regarding intervention adherence.

Data were collected in two waves with eight participants in each wave. Notably, some changes to study procedures and intervention delivery were made between waves to address concerns raised by participants in exit interviews. Specifically, wave one participants reported difficulties remembering the information provided during the orientation call; as such, in wave two, participants received a video tutorial and digital user guide via e-mail reviewing these details. Furthermore, an error in the eHealth engagement scale, where questions were formatted as rank order questions rather than Likert rating scales, was rectified. Finally, wave one participants reported frustration with the requirement to complete a tool (practice activity) within ASCENT multiple times before moving to the next lessons; therefore, this repetition requirement was removed for wave two participants.

#### 2.4.2. Measures

##### 2.4.2.1. Acceptability of Study Procedures

An investigator-developed 10-item survey was used to collect self-report data on the acceptability of study procedures. Participants were asked *“How satisfied were you with each of the following aspects of the study?”* with items including *“Completing the screening survey and informed consent form,” “The length of the follow-up assessments” and “Your interactions with the study staff.”* Participants responded to each item by choosing from 4 options: (1) *Not at all satisfied*; (2) *A little satisfied*; (3) *Somewhat satisfied*; and (4) *Very Satisfied*. An average acceptability rating was calculated for each participant. To proceed with the full factorial trial, we required that at least 68% participants have an average rating ≥ 3, indicating the procedures for the full factorial trial will be acceptable.

##### 2.4.2.3. Feasibility of Study Procedures

Feasibility was evaluated using recruitment rate (number enrolled/number successfully contacted) and retention rate (number of participants completing the follow-up assessment/number enrolled). In order for the full factorial trial to be considered feasible, we will require a minimum recruitment rate of 15% and a minimum retention rate 11/16, or 68% (21).

##### 2.4.2.3. Intervention Adherence

Data on intervention adherence was collected directly via the ASCENT platform. For each lesson participants accessed, the number of pages viewed was automatically collected; a completion percentage for each lesson was then calculated (number of pages viewed in lesson/total number of pages in lesson). A lesson was considered “started” if the participant viewed > 0% of pages and < 100% of pages; a lesson was considered “completed” if the participant viewed 100% of the pages in the lesson.

Participants also completed two investigator-developed self-report items regarding feasibility of intervention adherence. The first question asked, *“How difficult was it to complete all of the lessons in ASCENT over the course of 6 weeks?”* with response options including *Very Difficult, Somewhat Difficult, Somewhat Easy*, and *Very Easy.* The second question asked, *“How realistic was it to complete all of the lessons in ASCENT over the course of 6 weeks?”* with response options including *Very Realistic, Somewhat Realistic, Somewhat Unrealistic*, and *Very Unrealistic.*

##### 2.4.2.4. PROMIS Depression CAT

The PROMIS Depression Computer Adaptive Test (CAT(31)) was administered as this will be the primary outcome measure used in the full factorial trial. Here, we report only baseline levels of depressive symptoms in order to characterize the sample, though this measure was administered at baseline, weekly during the intervention period, and at follow-up. This validated measure assesses self-reported symptoms of depression (e.g., feelings of sadness, worthlessness, hopelessness, and loss of interest), with items asking participants to reflect on the past seven days. A Likert-scale, ranging from 1 = *Never* to 5 = *Always*, was utilized. The adaptive format tailors the test to each participant by selecting follow-up questions based on previous responses, which allows for fewer items to be administered while maintaining measurement precision. Scores are generated as standardized T-scores (*M* = 50, *SD =* 10), with high scores reflecting more severe depressive symptoms. Typically, T-scores under 55 are considered within normal limits, while scores of 55-59.9 indicate mild depressive symptoms, scores of 60-69.9 indicate moderate depressive symptoms, and scores of 70 or greater indicate sever depressive symptoms.

##### 2.4.2.5. Exit Interviews

Following the intervention period, participants were asked to complete a 45–60 minute semi-structured exit interview to gain insight into their experience with the study procedures and using ASCENT. Interviews were conducted by KMI, a clinical psychologist with prior experience conducting semi-structured interviews, or doctoral students trained by her to complete the interviews (RG, LW, and RA). In this paper, we present only results related to feedback on the study procedures. This was typically in response to the first four questions of the interview, which included a broad question about their overall experience, a question about their experience completing the assessments, a question about the automated reminders they received regarding completing their assessments, and a question about the monetary incentives provided for assessment completion. In addition to the initial question, interviewers utilized relevant probes to obtain additional information as needed, depending on the level of detail provided in the participant’s initial response.

### 2.5. Data Analysis

#### 2.5.1. Quantitative Analysis

Descriptive statistics were used to summarize participant demographics and clinical characteristics. A mean acceptability rating was calculated for each participant across the 10 items of the investigator-developed acceptability questionnaire. The proportion of participants with a mean acceptance score ≥ 3 out of those who completed the questionnaire was calculated and compared to our a priori goal of 68%. Similarly, recruitment rate (number enrolled/number successfully contacted) and retention rate (number of participants completing the follow-up assessment/number enrolled) were calculated and compared to a priori minimums (15% and 68% respectively). Finally, intervention adherence was calculated as the number of lessons started and the number of lessons completed out of the number of lessons assigned (which ranged from 6 to 30, depending on the number of components received).

#### 2.5.2. Qualitative Analysis

De-identified interview transcripts underwent qualitative analysis to better understand potential reasons for sub-optimal acceptability of any aspect of the study’s procedures. The Rapid and Rigorous Qualitative Data Analysis (RADaR) technique (32) was employed in two rounds; each round included qualitative data from seven different participants from both waves of data collection. The RADaR process uses five steps to analyze the data. First, study staff ensured all transcripts were formatted similarly (Step 1) and organized the transcripts in an all-inclusive Excel Phase 1 data table (Step 2). After this phase, 2–3 study staff members independently determined which quotes addressed the research question: *What changes do we need to make to study procedures to ensure their feasibility and acceptability?* Sentiments from the participants were only included if the response pertained to this specific question. Once this step was completed independently, the study staff members met as a group to discuss and reach a consensus on if the quote aligned with the specified research question (Step 3). Quotes that aligned with the specified research question produced the Phase 2 data table. The Phase 2 data table was then further refined by creating codes (Step 4). Lastly, a coding tree was created based on established codes to visualize the data (Step 5).

## 3. Results

### 3.1. Participant Demographic & Clinical Characteristics

A complete summary of participant demographics is available in Table 1. Most enrolled participants were emerging adults aged 18–25 (n = 8, 50%) rather than adolescents aged 15–17 (n = 1, 6.2%) or young adults aged 26–39 (n = 7, 44%). The mean age of enrolled participants at study screening was 26 years of age (S*D* = 7.1). Participant’s average age at cancer diagnosis was 22 years of age (*SD* = 6.5) and average time since active cancer treatment was 29 months (*SD* = 18).

Our sample was slightly more female (n = 9, 56%) than male (n = 7, 44%). The majority of our sample was White (n = 10, 63%), non-Hispanic (n = 14, 88%), and heterosexual (n = 10, 63%). Half of our sample had at least a college degree (n = 8, 50%). Additionally, most of our sample was single (n = 12, 75%) with no children (n = 12, 75%). Nearly one third of our sample had an annual household income of less than $25,000 (n = 5, 31%).

Our sample was quite diverse in terms of diagnosed cancer type and included participants with thyroid cancer being the most commonly reported diagnosis (n = 4, 25%) followed by brain, breast, leukemia, and non-Hodgkin’s lymphoma (all ns = 2). Participants tended to receive surgery (n = 13, 81%), radiation treatment (n = 9, 56%), and/or IV chemotherapy (n = 8, 50%) in their treatment regimen. Most participants were treated in adult cancer treatment facilities (n = 10, 63%).

At study screening, participant’s average score on the PROMIS-Depression CAT was 57 (*SD* = 9.2). Six participants (38%) scored within normal limits indicating sub-clinical levels of depressive symptoms, while four (25%) scored in the mild depression range and six (38%) scored in the moderate depression range. A two-sided independent samples t-test indicated that there was not a statistically significant difference in baseline depression scores between participants who completed the follow-up assessment and participants who did not (*t* = 1.9, *p* = 0.08).

Of the 16 participants that enrolled in the study, 12 completed the Week 6 follow-up assessment which included self-report data relevant to our outcomes. Of note, 12 participants also completed an exit interview with a study staff member at the end of the intervention period; however, one participant completed the follow up assessment but did not complete an exit interview, while one participant completed an exit interview but did not complete the follow up assessment. As such, the qualitative results only utilized data from the 12 participants that completed the exit interview (see Table 2 for a detailed account of the study procedures completed by each participant).

### 3.2. Primary Outcome: Acceptability of Study Procedures

#### 3.2.1. Quantitative Findings

Mean acceptability scores were calculated from the follow up assessment for each participant that completed it (n = 12). Overall, participants were somewhat to very satisfied with study procedures (*M* = 3.57, *SD* = 0.39, 95% CI = 3.32, 3.82). 11/12 participants (91.67%) had a mean acceptability score ≥ 3, and the lowest mean acceptability score was 2.9. Participants appeared to be most satisfied with aspects of study procedures that involved connecting with the study team; their ratings were highest on items related to the orientation call, reminders to complete assessments, and their interactions with study staff (All *Ms* = 3.92, All *SDs* = 0.29, All 95% CIs = 3.74, 4.10; see Table 3 for item-level means).

Participants were least satisfied with the length of the baseline assessment (*M* = 3.08, *SD* = 0.79, 95% CI = 2.58, 3.58), length of the weekly assessment (*M* = 3.08, *SD* = 0.90, 95% CI = 2.51, 3.65), and length of the follow-up assessment (*M* = 3.33, *SD* = 0.79, 95% CI = 2.83, 3.83). In sum, participant ratings suggest that study procedures for the future full factorial trial will be acceptable, but that some participants felt assessments were too long.

#### 3.2.2. Qualitative Findings

Qualitative analysis using the RADaR technique offered additional findings that helped our team characterize areas of sub-optimal acceptability (32). Participants not only had concerns about the length of the assessments, but also survey administration more broadly. Specifically, participants identified concerns and potential solutions related to clarity of items and response options, formatting, frequency and timing of assessment administration, length, indicators of progress on assessments, repetitiveness, and potential additions to the assessment (Table 4).

##### 3.2.2.1. Clarity

Participants found instructions for validity check questions aimed at confirming participant’s attention to be confusing. For example, one item stated “To answer this question, please choose option number four” on an assessment with item options labeled from 0 to 5. Participants were unsure whether to choose the response that was fourth from the left, or the response that was labeled as “4.” In addition, wave one participants identified an error in the programming of the eHealth Engagement Scale in which they were only allowed to choose each response once, when they needed the ability to choose multiple responses. This technical error was resolved prior to the start of wave two. Wave one participants were prompted to complete the impacted assessment again.

##### 3.2.2.2 Formatting

Regarding formatting, participants found that completing assessments on a mobile device resulted in occasional display errors (e.g., options did not perfectly align with headers), making it difficult to provide accurate responses. Such a finding is particularly important to consider in our study given that participants were able to opt into receiving reminders to complete assessments via text messages, which may have increased the likelihood of completing assessments on mobile devices.

##### 3.2.2.3. Frequency and Timing of Assessment Administration

Frequency and timing of weekly assessments were commonly brought forth as areas for improvement. Participants suggested reducing the frequency of weekly assessments to reduce participant burden. Additionally, participants’ weekly assessment due dates were based on the date they completed the orientation call. Thus, some participants received assessments in the middle of the week, which at times made it difficult to consider events and feelings they experienced “in the past week.” Participants suggested that timing weekly surveys to be completed at the end of the week may make them less cognitively taxing.

##### 3.2.2.4. Survey Length, Repetitiveness, and Progress

Concerning length and repetitiveness, qualitative findings confirmed that users felt that assessments at all time points were lengthy and should be streamlined. Participants suggested removing questions that felt repetitive (e.g., “In the last seven days, I felt depressed” and “In the last seven days, I felt sad”), as answering what seemed to be the same question several times increased their perception that the assessment was too long. Other suggestions participants made for addressing concerns regarding perceived assessment length included having an indicator of progress to help them know how many questions remain and providing the option to save their progress and return later (particularly for the lengthier baseline and follow-up assessments).

##### 3.2.2.5. Suggested Additions to Surveys

Finally, alongside concerns about length, participants had some suggestions for what should be added to the assessments. Specifically, they suggested including items regarding completion of the lessons as planned, as well as items that would foster self-reflection about their experience in the study.

### 3.3. Secondary Outcome: Feasibility of Study Procedures

**Figure 2:**
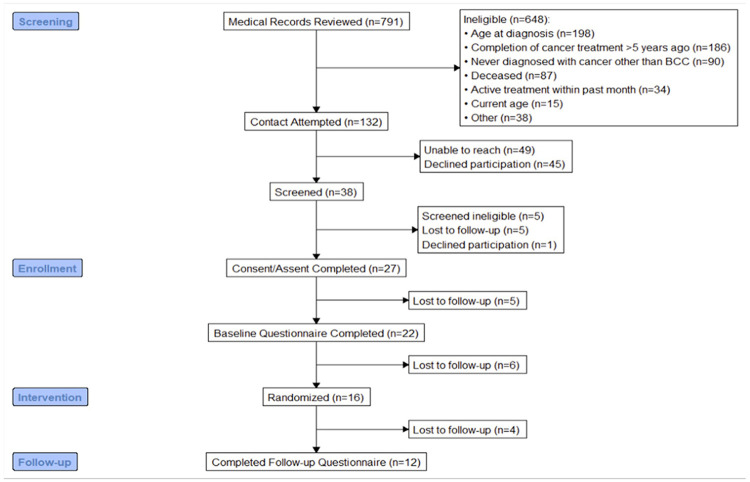
CONSORT Diagram

We initially identified 847 potentially eligible patients (93 adolescents, 245 emerging adults, and 509 young adults) via a report from our institution’s EMR. Later in the study we obtained a separate report from the cancer registry that identified 617 potentially eligible patients (25 adolescents, 116 emerging adults, 476 young adults). However, there was significant overlap between these two methods of identifying potentially eligible patients. Over the course of the study, we completed a detailed review of 791 EMRs. Throughout this process, 648 patients were determined to be ineligible due to age at diagnosis outside of the eligible range (n = 198, 30.6%), completion of active cancer treatment more than 5 years ago (n = 186, 28.7%), never being diagnosed with cancer or only having a diagnosis of basal cell carcinoma (n = 90, 13.9%), being deceased (n = 87, 13.4%), current receipt of active treatment within the past month (n = 34, 5.2%), current age (n = 15, 2.3%), or another reason (n = 38, 5.9%) such as need for an interpreter or documented diagnosis of a severe or persistent mental illness noted in our exclusion criteria. The remaining 143 patients (17 adolescents, 59 emerging adults, and 67 young adults) were deemed potentially eligible, prompting contact with their physician to obtain approval to contact the patient for recruitment purposes. Approval for recruitment was obtained for 132 potentially eligible patients at which point patients were contacted by study staff up to 5 times via patient portal message, e-mail, and/or phone. Of those we attempted to reach regarding screening, study staff successfully contacted 83 individuals. Of those, 38 completed study screening, and 30 were eligible. Ultimately, 27 individuals consented/assented to participate in the study, 22 completed the baseline questionnaire, and 16 completed the orientation call at which point they were considered enrolled. Of the 16 enrolled participants, 9 completed all weekly assessments, 12 completed the follow-up questionnaire, and 12 completed the exit interview. This is summarized in Fig. 2.

Our recruitment rate was 19% (16 enrolled of 83 successfully contacted), surpassing the minimum threshold of 15%. The retention rate was 75% (12 of 16 enrolled participants completed the six-week follow-up), exceeding the a priori criterion of 68%. Taken together, these findings indicate that recruitment and retention strategies used in the pilot study are feasible. However, qualitative feedback highlighted several areas for refinement of study procedures (Table 4). Participants reported that recruitment materials would be strengthened by including more specific information about compensation and by making study details accessible through official institutional websites to improve legitimacy. Several participants also noted that recruitment emails were occasionally filtered to spam, underscoring the importance of using multiple modalities for recruitment contacts. Regarding retention, participants suggested that reminders for survey completion should emphasize deadlines and expected completion time, as well as provide additional late-day reminders to improve response rates. Overall, while recruitment and retention strategies were acceptable, these suggested adjustments may further enhance the feasibility of a larger trial.

### 3.4. Exploratory Outcome: Intervention Adherence

On average, participants started 45% of the ASCENT lessons they were assigned, meaning they viewed at least one screen in a lesson they were expected to complete during the intervention period. Similarly, participants completed 42% of the content that they were assigned on average, meaning they viewed all screens of a lesson they were instructed to complete during the intervention period. There was a wide range of completion rates among participants (e.g., one participant completed 3% of their assigned content while two completed 100% of their assigned content).

Further, we examined participants’ perceptions of difficulty and feasibility of completing assigned content. Interestingly, participants did not find it difficult nor easy to complete their assigned content. The mean score for the item, “How difficult was it to complete all of the lessons in ASCENT over the course of 6 weeks?” was 2.8 (*SD* = 1.1), which falls between the somewhat difficult (2) and somewhat easy (3) ratings. No statistically significant differences in this rating were observed based on the number of lessons assigned. Participants also reported that the amount of content they were assigned to complete was realistic, with an overall mean score on the item, “How realistic was it to complete all of the lessons in ASCENT over the course of 6 weeks?” being 3.2 (*SD* = 0.83), which falls between somewhat and very realistic. Notably, a statistically significant difference in this rating was observed between participants who were assigned 24 or more lessons (n = 4, *M* = 2.5, *SD* = 1.0, 95% CI = 0.91. 4.09) and participants who were assigned 18 or fewer lessons (n = 8, *M* = 3.5, *SD* = 0.53, 95% CI = 3.06, 3.94; *t*(10) = 2.3, *p* = 0.04). In summary, if participants started a lesson, they almost always completed it. However, there was a wide range of adherence to assigned intervention components. Participants also viewed completing their assigned content during the intervention period as a realistic goal, but they did not find it to be particularly easy nor difficult.

## 4. Conclusions

We achieved the study’s defined objectives to (1) assess the acceptability and feasibility of study procedures for a full factorial trial examining a digital depression self-management tool for AYACS and (2) examine intervention adherence. We found that procedures were acceptable, with 92% of participants indicating they were somewhat or very satisfied with procedures overall. Item-level acceptability ratings indicated participants were least satisfied with the length of assessments. This suggests that the procedures for the planned full factorial trial will be acceptable. Qualitative data supported these findings and revealed additional procedural concerns related to survey administration; participants desired improved survey formatting, greater clarity in the survey items and response options, and modified timing for receiving and completing surveys. Participants also provided potential solutions to improve the acceptability of survey administration procedures, such as including items about completion of ASCENT lessons as planned and self-reflection focused items for participants to consider their experiences being part of the study. Similarly, recruitment and retention rates were above a priori benchmarks, indicating that the future full factorial trial will be feasible. Participants suggested ways to fine-tune existing recruitment and retention strategies such as providing additional information about the study online, utilizing multiple modalities for recruitment, and clarifying study procedures. Finally, we explored participant adherence to completion of assigned intervention content. On average, participants completed under half of the content they were assigned (42%). While participants indicated that it was realistic to complete assigned content, they found it somewhat difficult to do so in the given study timeframe, especially if they were assigned to four or more study components (equivalent to 24 or more lessons). Overall, our data revealed important areas for improvement and potential adjustments to study procedures to enhance recruitment, retention, and adherence to ASCENT. These findings contribute to the dearth of literature on digital mental health interventions for AYACS while also addressing gaps in understanding the feasibility and acceptability of complex research procedures to evaluate such interventions.

### 4.1. Comparison with Past Research

While past research has evaluated the feasibility and acceptability of digital health and psychosocial interventions for AYACS (33-36), *acceptability of study procedures* of intervention trials for AYACS is less understood. Corke et al. (2025) and Wurz and Brunet (2019) examined acceptability of study procedures in their pilot rehabilitation and physical activity intervention programs for AYACS, respectively (37, 38). Similar to our qualitative and quantitative results supporting procedural acceptability, qualitative interview findings from these studies revealed that participants found study procedures widely acceptable (37, 38). Our investigation offers robust findings and support for study procedures, given the mixed-methods nature of our acceptability data. Not only did our qualitative and quantitative findings inform our future full factorial trial, but the results also contribute to crafting a more comprehensive understanding of AYACS’ preferences for engaging in digital intervention research. Further, the findings provide support for the acceptability of conducting trials with complex designs in a hard-to-reach population.

Study feasibility, which can be measured by recruitment and retention rate, has been more commonly evaluated in AYACS digital intervention research. Salsman et al. (2023)’s pilot feasibility trial of a self-guided eHealth positive emotions intervention for AYACS reported a recruitment rate of 20% and retention rates of 61% at eight weeks and 12 weeks (11). Considering that the current study’s recruitment rate was 19% and its retention rate was 75%, our feasibility metrics were comparable to those found in trials of interventions delivered in a similar manner. Other digital intervention studies for AYACS, though different in their procedural and methodological approaches from the current study’s, can be compared to our findings as well. For example, Zhang et al. (2023)’s pilot feasibility study of a technology and coach-assisted cognitive behavioral therapy (tCBT) for depression in AYACS demonstrated a 35% recruitment rate and an 80% retention rate (17). Similarly, Tutelman et al. (2024)’s pilot feasibility and acceptability study of an online group psychotherapy intervention for AYACS had a 53% recruitment rate (15). Although the current study’s recruitment rate was comparatively lower, there were differences in recruitment strategies and intervention design which may have contributed to disparities. Both Zhang et al. (2023) and Tutelman et al. (2024) recruited AYACS in active cancer treatment and those who had completed treatment, unlike our study which only recruited AYACS between one month and five years post treatment (15, 17). Our study used a multi-step recruitment method involving direct patient communication, while Tutelman et al. (2024) and Zhang et al. (2023) recruited participants through referrals and/or electronic flyer distribution (15, 17). Intervention wise, Zhang et al. (2023) and Tutelman et al. (2024) integrated scheduled direct interactions between participants and its study team members (15, 17). We intentionally did not incorporate similar contacts with participants in an effort to foster participant independence in using ASCENT given our goal of optimizing procedures for a self-management tool. Despite our study’s lack of direct contact with patients during the intervention period, our study’s retention rate (75%) was quite similar to Zhang et al’s retention rate of 80%, suggesting that other aspects of study procedures and/or the ASCENT intervention may have supported participant retention (17). Altogether, our study’s feasibility findings address a gap in knowledge of the feasibility of AYACS digital intervention studies and generally align with available insights.

In terms of adherence to the ASCENT intervention, on average, pilot participants completed 42% of the lessons they were assigned. This is somewhat lower than metrics of adherence in self-guided digital interventions for AYACS and AYAs reported in other studies. For example, Salsman et al. (2023) also targeted AYACS and calculated adherence to EMPOWER as the number of intervention sessions completed (11). Participants completed four out of five intervention sessions on average and 82% of participants completed three or more intervention sessions. Comparably, Miller et al. (2023) measured adherence to a five-module mobile app intervention for depression (Spark) in 30 AYAs based on module completion (39). Half of participants completed all five modules of the Spark intervention after five weeks (39), while only 12.5% of participants in ASCENT completed all assigned modules after six weeks. Notably, EMPOWER and Spark had five lesson sessions/modules; in contrast, participants in the ASCENT pilot were assigned to complete 6 to 30 lessons across 1 to 5 modules over the course of six weeks, depending on the condition they were assigned to. Indeed, participants in the present study found it somewhat difficult to complete their assigned content within the study timeframe, especially if they were assigned to four or more study components (equivalent to 24 or more lessons). Thus, this disparity in adherence rates may be a function of the *amount* of content participants were asked to complete, rather than the intervention itself. Conducting a full-factorial trial of ASCENT will help us to determine which components should be included in an optimized version of the tool, ensuring that unnecessary aspects of the intervention are removed. We expect that this will ultimately enhance intervention adherence.

### 4.2. Strengths

The present study has two key strengths: (1) it provided a systematic evaluation of the feasibility and acceptability of study procedures of a complex trial design for a hard-to-reach population and (2) it employed mixed-methods to do so, providing rich insights into factors that impacted study feasibility and acceptability. Past investigations of psychosocial interventions for AYACS have predominantly employed single-arm designs or pilot randomized controlled trial designs with two arms. While some digital interventions for AYACS are now being tested using complex full factorial designs (40, 41), existing literature lacks examination of the feasibility and acceptability of complex study procedures required by full factorial trials that aim to optimize such interventions. Our study addresses this critical gap by systematically evaluating the feasibility and acceptability of the procedures themselves in the context of a more complex design with 16 experimental conditions. This method provides a nuanced understanding of procedural elements necessary for ensuring the success of complex large-scale trials for a unique and underserved population. The novelty of the study’s approach is particularly important for AYACS, who have historically been a difficult population to recruit and retain in clinical and patient-reported outcome research (42-44).

The current study employed a mixed-methods design by analyzing quantitative and qualitative data to capture both measurable participant-reported outcomes and participant perspectives on study procedures. In line with Aschbrenner et al. (2022), feasibility domains such as recruitment, randomization, retention, assessment procedures, implementation resources, intervention delivery, adherence, and acceptability can be examined using both numerical indicators and participant narratives to strengthen conclusions and guide future trial design (45). From study conception, our rationale for a mixed-methods approach was to achieve a comprehensive and contextually grounded evaluation that extends validity beyond what a single method could provide (46). By addressing feasibility questions with input from both quantitative metrics and qualitative sentiments, the study provided greater insight into how participants experienced and interpreted their participation in the study. Mixed-methods were particularly relevant in this context, as they enabled us to incorporate participants’ perspectives directly into the evaluation of the pilot, prioritizing participant voices in developing a tool that is optimized and responsive to the needs of AYACS.

### 4.3. Limitations

Despite the study’s strengths, our research is not without shortcomings. This investigation tested procedures for a full factorial trial of ASCENT at a rural institution using a small sample size. Although our findings suggest that study procedures will be acceptable and feasible in a future full factorial trial, results may not be generalizable to other settings. For example, other institutions may have different requirements and restrictions for contacting patients compared to the procedures we investigated. Additionally, the study sample was mostly White, non-Hispanic, heterosexual, single individuals without children who have attended at least some college. Further, the sample only included one adolescent (aged 15–17) participant, which may be the result of both a smaller recruitment pool and additional requirements during participant recruitment for adolescents (i.e., obtaining caregiver permission) to participate. Further strategies will be employed in the full factorial trial to promote adolescent recruitment. Specifically, we aim to strengthen connections between our study team and local pediatric oncologists and hospital psychosocial team members to promote referrals. This is consistent with findings from the field of implementation science which suggest that physician champions play a key role in ensuring the success of initiatives that require practice changes (47). We are also exploring alternative recruitment sources, such as the Children’s Oncology Group (COG)’s Project: Every Child Childhood Cancer Registry to reach a larger pool of younger AYACS (48).

### 4.4. Conclusion

The findings of our pilot study bolster current literature on digital interventions for depressive symptoms in AYACS and revealed that the study procedures for our full factorial trial of ASCENT are acceptable and feasible. In addition, our team garnered valuable insight into what participants felt most satisfied with in our study procedures (e.g., reminders to complete assessments, interactions with study staff, study orientation call) and what they were less satisfied with (e.g., length of assessments, assessment formatting, pre-enrollment education on the study). The mixed method approach unveiled a wide range of participant approvals and disapprovals of study procedures that will ultimately be used to adjust procedures in a full factorial trial of ASCENT. These findings are not only crucial to our planned full factorial trial of ASCENT but may also inform procedure development for future investigations of digital health tools tailored to AYACS that require evaluation via complex trial designs.

## Figures and Tables

**Table 1:** Baseline demographic and clinical characteristics of study participants.

Characteristic		Enrolled (N = 16)		Completed (N = 12)		Lost to Follow-up (N = 4)	
		Mean	(SD)	Mean	(SD)	Mean	(SD)
Age at screening		26	(7.1)	26	(6.9)	25	(7.4)
Age at cancer diagnosis		22	(6.5)	22	(5.8)	23	(8.0)
Months since completion of cancer treatment		28	(18)	29	(18)	23	(13)
Baseline PROMIS-Depression CAT		57	(9.2)	55	(9.4)	64	(3.5)
Characteristic	Group	Enrolled (N = 16)		Completed (N = 12)		Lost to Follow-up (N = 4)	
		N	(%)	N	(%)	N	(%)
Sex							
	Female	9	(56%)	6	(50%)	3	(75%)
	Male	7	(44%)	6	(50%)	1	(25%)
Race							
	Mixed Racial Background	4	(25%)	2	(17%)	2	(50%)
	Other Race	2	(13%)	1	(8.3%)	1	(25%)
	White/Caucasian	10	(63%)	9	(75%)	1	(25%)
Ethnicity							
	Hispanic	2	(13%)	1	(8.3%)	1	(25%)
	Non-Hispanic	14	(88%)	11	(92%)	3	(75%)
Sexual Orientation							
	Bisexual	3	(19%)	2	(17%)	1	(25%)
	Gay	2	(13%)	2	(17%)	0	(0.0%)
	Heterosexual	10	(63%)	7	(58%)	3	(75%)
	Pansexual	1	(6.3%)	1	(8.3%)	0	(0.0%)
Level of Education							
	Less than High School	1	(6.3%)	1	(8.3%)	0	(0.0%)
	High School Diploma or Equivalent	1	(6.3%)	0	(0.0%)	1	(25%)
	Some College	6	(38%)	4	(33%)	2	(50%)
	College Graduate	7	(44%)	6	(50%)	1	(25%)
	Graduate Degree	1	(6.3%)	1	(8.3%)	0	(0.0%)
Marital Status							
	Divorced	2	(13%)	1	(8.3%)	1	(25%)
	Living with Partner	1	(6.3%)	0	(0.0%)	1	(25%)
	Married	1	(6.3%)	1	(8.3%)	0	(0.0%)
	Single, never married	12	(75%)	10	(83%)	2	(50%)
Have Children							
	Yes	4	(25%)	3	(25%)	1	(25%)
	No	12	(75%)	9	(75%)	3	(75%)
Household Income							
	Less than $25,000	5	(31%)	4	(33%)	1	(25%)
	$25,000 to $34,999	2	(13%)	0	(0.0%)	2	(50%)
	$35,000 to $49,999	2	(13%)	1	(8.3%)	1	(25%)
	$50,000 to $74,999	2	(13%)	2	(17%)	0	(0.0%)
	$75,000 to $99,999	1	(6.3%)	1	(8.3%)	0	(0.0%)
	$100,000 or more	2	(13%)	2	(17%)	0	(0.0%)
	Don’t know/Prefer not to answer	2	(13%)	2	(17%)	0	(0.0%)
Diagnosed Mental Health Condition							
	Yes	12	(75%)	8	(67%)	4	(100%)
	No	3	(19%)	3	(25%)	0	(0.0%)
	Unsure	1	(6.3%)	1	(8.3%)	0	(0.0%)
Current Mental Health Treatment^a^							
	Individual therapy	2	(13%)	0	(0.0%)	2	(50%)
	Medication	8	(50%)	7	(58%)	1	(25%)
	None	6	(38%)	5	(42%)	1	(25%)
Cancer Type(s)^a^							
	Brain	2	(13%)	1	(8.3%)	1	(25%)
	Breast	2	(13%)	1	(8.3%)	1	(25%)
	Hodgkin’s Lymphoma	1	(6.3%)	1	(8.3%)	0	(0.0%)
	Leukemia	2	(13%)	2	(17%)	0	(0.0%)
	Myeloma	1	(6.3%)	1	(8.3%)	0	(0.0%)
	Non-Hodgkin’s Lymphoma	2	(13%)	2	(17%)	0	(0.0%)
	Sarcoma	1	(6.3%)	1	(8.3%)	0	(0.0%)
	Testicular	1	(6.3%)	1	(8.3%)	0	(0.0%)
	Thyroid	4	(25%)	2	(17%)	2	(50%)
Cancer Treatment Type(s)^a^							
	Surgery	13	(81%)	9	(75%)	4	(100%)
	Radiation	9	(56%)	7	(58%)	2	(50%)
	IV Chemotherapy	8	(50%)	7	(58%)	1	(25%)
	Stem Cell Transplant	1	(6.3%)	1	(8.3%)	0	(0.0%)
	Immunotherapy	1	(6.3%)	1	(8.3%)	0	(0.0%)
	Hormone Therapy	5	(31%)	2	(17%)	3	(75%)
Type of Cancer Treatment Facility^a^							
	Children’s Hospital	6	(38%)	4	(33%)	2	(50%)
	Adult Hospital/Academic Medical Center	10	(63%)	9	(75%)	1	(25%)
	Specialized Cancer Care Clinic	2	(13%)	2	(17%)	0	(0.0%)
	Private Practice	1	(6.3%)	1	(8.3%)	0	(0.0%)

a Participants could select multiple responses to these items, so percentages may total to greater than 100%

**Table 2:** Study Procedures Completed by Participants.

Survey	Participant ID																
	1	2	3	4	5	6	7	8	9	10	11	12	13	14	15	16	Percent Complete
**Week 1**	X	X	X		X	X			X		X	X	X	X	X	X	75%
**Week 2**	X	X	X		X	X	X	X	X		X	X	X	X	X	X	88%
**Week 3**	X	X	X		X	X	X	X	X			X	X	X	X	X	81%
**Week 4**	X	X	X		X	X	X	X	X	X		X		X	X		75%
**Week 5**	X	X	X		X	X	X	X	X	X		X		X	X		75%
**Week 6 Follow up**	X	X	X		X	X	X	X	X	X		X		X	X		75%
**Exit Interview**	X	X	X		X	X	X	X	X	X	X	X		X	X		81%

**Table 3:** Acceptability of Study Procedures

Item Number & Content	Mean	SD	Median	Min	Max
1. Being provided information about the study before deciding to join	3.75	0.62	4	2	4
2. Completing screening survey and informed consent form	3.75	0.45	4	3	4
3. Length of baseline assessment	3.08	0.79	3	2	4
4. Orientation call	3.92	0.29	4	3	4
5. Reminders to complete assessments	3.92	0.29	4	3	4
6. Frequency of weekly assessments	3.42	0.67	3.5	2	4
7. Length of weekly assessments	3.08	0.90	3	2	4
8. Length of follow-up assessment	3.33	0.65	3	2	4
9. Interactions with study staff	3.92	0.29	4	3	4
10. Participating in the study overall	3.50	0.52	3.5	3	4

**Table 4:** Themes, sub-themes, and exemplar quotes derived from exit interviews.

Theme	Sub-theme	Exemplar Quote(s)
Surveys	Clarity of Items & Response Options	**Validity Checks: “**Then there was one thing I ran into quite a few times, and I did want to mention this just in case, my answers didn’t seem like they were lining up correctly. Throughout some of the number answering ones where it was like “answer one to four”, You cross a question that says like, “choose answer number 4, right? So you know, you’d bubble in #4 at that point, right? But when there is 0-1-2-3-4-5 like the 4th one is actually 3. I was like, “do they want me to bumble in the fourth bubble or number 4? You know what I mean? So that was just something that kind of confused me a little bit, but I just picked one and rolled with it.” *(26-year-old Female Thyroid Cancer Survivor)* **Questionnaire Items**: “There were some of the questions that based off of like the one through 5 or like kind of like what each one read could be a little misleading because it almost didn’t like implies a double negative on some of the answers. Um so there were a couple that I had to like reread to make sure that I was answering correctly or truthfully. Um but overall, I think it not only happened, maybe a handful of times, like maybe once or twice each survey. So it wasn’t very frequently, but um I think it was I can’t remember the exact question, but it was I think in the last seven days were you not X,Y, and Z and then the the options were not like nearly ever.” *(35-year-old Male Thyroid Cancer Survivor)* **Response Entry Error**: “I did notice on the follow up assessment, there was a table that had you selecting things, and you couldn’t select an option for everything. It worked like the other tables did for the rest of the assessments, but it had a series of things. You had to answer just with those radio buttons, and you could only choose one of the options for each row. After you have chosen them once, if you made the same choice in that same column again for a later option, it would just replace it. It would remove it from the previous time that you made that choice. So you could only choose that option once for anything in that column. I dont think it was intentional. Because why would you have all those options for each row if you literally couldn’t choose an option in every row?” *(34-year-old Male Hodgkin Lymphoma Survivor)*
	Formatting	“I don’t know if it was just the way the format ends up appearing on my browser sometimes, but I feel like there are a lot of times where it would, depending on if I tried to complete it on my phone or on a browser, it would kind of scrunch the headers for the tables that you had for like the multiple choice sections.” *(34-year-old Male Hodgkin Lymphoma Survivor)*
	Frequency & Timing of Administration	**Frequency**: “Um but maybe like could either even maybe extending instead of like a weekly, maybe do a week and 1/2 for each one? So like maybe every three weeks you complete two sections. So maybe it extends the study a little bit longer but then gives a little bit uh people have a little extra breathing room. If you want to keep it kind of standard.” *(35-year-old Male Thyroid Cancer Survivor)* **Timing**: “I feel like it was a little off when it was every Wednesday ’cause. I feel like that was kind of like in the middle of the week. I guess compared to like the end of each week. So. Sometimes, like answering, I mean obviously I would answer for that specific like seven days, but. In my head I’m thinking like this week so sometimes answering stuff um might have been off for me, I guess.. Or like Thursday, or I guess sometimes I didn’t get a chance or I wouldn’t get the chance to go answer it until Thursday. But yeah, more, more kind of towards the end of the week, yeah.” *(30-year-old Female Acute Lymphoblastic Leukemia Survivor)*
	Length & Repetitiveness	**Baseline Questionnaire**: “…especially since the intro survey I put off because I felt like it was, you know, too long. Like there was just a mountain of questions that I had to answer and I had very little time to complete it because I believe that when I was….uh had started that I was in the midst of completing my senior project or, you know, just getting into the thick of….Just all of the components surrounding my project. So I had to just prioritize my work. So yeah, I’d say that was a hurdle I had to overcome just trying to find time intervals to just complete these assessments.” *(22-year-old Male Sarcoma Survivor)* **Weekly Questionnaire**: “Um I mean, it [the weekly questionnaire] was like [on the] lengthier side.” *(24-year-old Female Non-Hodgkin Lymphoma Survivor)* **Follow-up Questionnaire**: “I feel like it could probably be shortened a bit, but I understand if you want certain data points and it looks like it’s the- I don’t know. Like a lot of them, say Vanderbilt at the bottom. So I don’t know if that was like- if it’s already like a built tool that you can’t really change up now…It was a little long though, and I’m still- I started this 25 minutes ago trying to get through the final one before this call, but I haven’t been successful at that. *(33-year-old Female Myeloma Survivor)* **Repetitiveness**: “And then sometimes, not repetitive is the word, but like I just didn’t see the difference, like it was like how many times do you feel happiness? How many times do you feel joy? How many times have you felt cheerful? How many times have you felt delighted? I’m just like, aren’t those all the same thing?” *(18-year-old Female Leukemia Survivor)*
	Progress	**Indicator**: “Right. So uh, I think I was just uh, I was really uh thrown off by the lack of maybe a completion marker at the bottom. I know that the survey said that it takes like 30 to 45 minutes to complete, but sometimes I either just uh think about questions or I just answer them rapidly. So really I just I think having a..just a progress bar at the bottom or something that would be helpful instead of just clicking to the next page and seeing a thank you for your responses.” *(22-year-old Male Sarcoma Survivor)* **Option to Save & Return**: “So possibly if there’s a way to maybe even do a login. I don’t know tech wise, how you’re doing it? If it’s like a unique, I’m assuming it’s a um unique URL for each user. So if it could maybe redirect to something that has like a uh pass through login or something. That could kind of associate it with that account or that person that’s doing the survey, possibly. I also didn’t try going back to the the URL more than once, so I wasn’t sure if it expired after a certain amount of time either.” *(35-year-old Male Thyroid Cancer Survivor)*
	Suggested Additions	**Questions about Completion of Intervention Content**: “I would also just like for there to be some like questions about the lessons for the weekly assessments, you know as just a reminder to that, I needed to complete my lessons each week as they were scheduled as I feel like that’s just a great way to keep up with them.” *(22-year-old Male Sarcoma Survivor)* **Questions to Foster Self-Reflection**: “So it could even, so maybe like if we response open section um for the user of saying like what was the like, Did you learn anything new about yourself during this time, because of your cancer diagnosis and in the training or something of that nature. Um so being able to kind of look at that and then kinda tie those different pieces together um could definitely help um others if they don’t have that self reflection either.” *(35-year-old Male Thyroid Cancer Survivor)*
Provision of information about study	Availability of Information Pre-Consent	“I do try to do some like research online because I work in tech. Try like you know that that things beforehand. So just trying to make sure that I could find as much about the study beforehand. So like anything publicly facing was really difficult to find um and very sparse for the information. Um so just for me working and you know trying to working in the tech industry, not wanting to get, you know scammed like with an e-mail um like trying to find some reputable sources for it was a little sparse. So I would say that would be the only thing that could be um updated a little bit more beforehand, so maybe if something could be on like the ECU’s website. About like even a quick blurb about what the study is or something a little bit easier to find because even if there was a link to the ECU website, um just being able to be on their domain about like this is an ongoing study could have been helpful.” *(35-year-old Male Thyroid Cancer Survivor)*
	Specificity	“I think some things that maybe could have been um a little more like specific… I guess like at first. At first, like I I I didn’t even realize it was like a paid thing or that like that. It was that but um. And I guess the um. What I was told, I feel like in the beginning. Was not um. Or I guess from what I read about it and then when I talked about it in like the beginning interview um It wasn’t exactly um as much as I thought.” *(30-year-old Female Acute Lymphoblastic Leukemia Survivor)*
Recruitment Contacts	Format	“Luckily I was actually going through my spam so that could be something to possibly look at in the future and saw the the e-mail from through there and was able to connect and um then was able to…learn a little bit about the study” *(35-year-old Male Thyroid Cancer Survivor)* “I feel like maybe adding a little call in there might be useful…So that’s where I see a problem coming in. Not much people check their e-mail, so they might not get the chance to see it. And participate in it….” *(18-year-old Male Brain Cancer Survivor)*
Survey Reminders	Content	**Deadline for Completion**: “I think maybe I just didn’t realize it needed to be done that day. Maybe that’s what it was. I think they come through e-mail and until I got the angry e-mail being like YOU DIDN’T DO IT YESTERDAY, um like I think it wasn’t maybe clear to me or I didn’t read the e-mail in full to say like it has to be done today, we want it done today….You know, or just like in bold or some something in that e-mail to be like please complete it today. Not like when you get around to it- today.” *(33-year-old Female Myeloma Survivor)* **Duration**: “Yeah, I mean it [expected amount of time to complete survey] might be something to add. So people know it’s not just something like I said, in the grocery store something you just pull up and click through, like if you’re doing it with integrity, you’re not just clicking through it to get it done.” *(18-year-old Female Leukemia Survivor)*
	Schedule	**Personalization**: “I can’t remember if this was done at the beginning of the survey, is maybe asking when the reminders you would prefer the reminder to be sent out. So like if I could have like chosen a time that would have been after work. Um that, and I think the majority of the time it came in during work, so. See it then, Like, forget about it and then go back to it. So if there’s an option possibly to schedule the time for for specific people.” *(35-year-old Male Thyroid Cancer Survivor)* **2nd Reminder**: “Yeah, because there was only, I think one reminder during the day like for text message, I think it would be like another reminder at night just to make sure they complete it or something but other than that…” *(19-year-old Male Non-Hodgkin Lymphoma Survivor)*

## Data Availability

The datasets supporting the conclusions of this article are available in the OSF repository, https://osf.io/asbj5/overview?view_only=98091fc6fbb941618501f5e0900664f8 .

## KEY MESSAGES ON FEASIBILITY

1. **What uncertainties existed regarding the feasibility?** We aimed to establish whether the recruitment and retention procedures for the planned full factorial trial would be feasible. This was uncertain given the low participation rates of AYACS in trials, the complex trial design, and the novelty of the intervention content.
2. **What are the key feasibility findings?** Recruitment and retention rates exceeded a priori benchmarks, suggesting the recruitment and retention procedures would be feasible in the larger trial. However, qualitative interviews provided insights regarding ways to further enhance feasibility.
3. **What are the implications of the feasibility findings for the design of the main study?** While recruitment and retention rates indicated the full factorial trial is feasible, the larger trial would likely benefit from fine-tuning the existing recruitment and retention strategies. Guided by qualitative insights, we aim to enhance recruitment and retention rates in the main study by reducing the length of assessments, providing additional information pre-enrollment, and adjusting the frequency of reminders to complete study procedures throughout the trial.

## References

[1] Page LL, Devasia TP, Mariotto A, Gallicchio L, Mollica MA, Tonorezos E. Prevalence of cancer survivors diagnosed during adolescence and young adulthood in the United States. JNCI J Natl Cancer Inst. 2025;djae250. doi:10.1093/jnci/djae250 PMC11884855 39383200

[2] National Cancer Institute. Cancer Stat Facts: Cancer Among Adolescents and Young Adults (AYAs) (Ages 15–39) [Internet]. 2025. Available from: https://seer.cancer.gov/statfacts/html/aya.html

[3] Miller KD, Fidler-Benaoudia M, Keegan TH, Hipp HS, Jemal A, Siegel RL. Cancer statistics for adolescents and young adults, 2020. CA Cancer J Clin. 2020;70(6):443–59. doi:10.3322/caac.21637 32940362

[4] Darlington AE, Wakefield CE, Van Erp LME, Van Der Graaf WTA, Cohn RJ, Grootenhuis MA. Psychosocial consequences of surviving cancer diagnosed and treated in childhood versus in adolescence/young adulthood: A call for clearer delineation between groups. Cancer. 2022;128(14):2690–4. doi:10.1002/cncr.34257 35579570

[5] Lang MJ, David V, Giese-Davis J. The Age Conundrum: A Scoping Review of Younger Age or Adolescent and Young Adult as a Risk Factor for Clinical Distress, Depression, or Anxiety in Cancer. J Adolesc Young Adult Oncol. 2015;4(4):157–73. doi:10.1089/jayao.2015.0005 26697266 PMC4684657

[6] Salsman JM, Garcia SF, Yanez B, Sanford SD, Snyder MA, Victorson D. Physical, emotional, and social health differences between posttreatment young adults with cancer and matched healthy controls. Cancer. 2014;120(15):2247–54. doi:10.1002/cncr.28739 24888335 PMC4121054

[7] Osmani V, Hörner L, Klug SJ, Tanaka LF. Prevalence and risk of psychological distress, anxiety and depression in adolescent and young adult (AYA) cancer survivors: A systematic review and meta-analysis. Cancer Med. 2023;12(17):18354–67. doi:10.1002/cam4.6435 37559504 PMC10523984

[8] Zhang A, Urban-Wojcik E, Seewald M, Zebrack B. Mental Health Trajectories Among US Survivors of Adolescent and Young Adult Cancer as They Age. JAMA Netw Open. 2025;8(5). doi:10.1001/jamanetworkopen.2025.11430 PMC12090028 40388164

[9] Yi JC, Syrjala KL. Anxiety and Depression in Cancer Survivors. Med Clin North Am. 2017;101(6):1099–113. doi:10.1016/j.mcna.2017.06.005 28992857 PMC5915316

[10] Rabin C, Simpson N, Morrow K, Pinto B. Intervention Format and Delivery Preferences Among Young Adult Cancer Survivors. Int J Behav Med. 2013;20(2):304–10. doi:10.1007/s12529-012-9227-4 22328444

[11] Salsman JM, McLouth LE, Tooze JA, Little-Greene D, Cohn M, Kehoe MS, An eHealth, positive emotion skills intervention for enhancing psychological well-being in young adult Cancer survivors: results from a multi-site, pilot feasibility trial. Int J Behav Med. 2023;30(5):639–50. 36890329 10.1007/s12529-023-10162-5 PMC10485177

[12] Campo RA, Bluth K, Santacroce SJ, Knapik S, Tan J, Gold S, A mindful self-compassion videoconference intervention for nationally recruited posttreatment young adult cancer survivors: feasibility, acceptability, and psychosocial outcomes. Support Care Cancer. 2017;25(6):1759–68. doi:10.1007/s00520-017-3586-y 28105523

[13] Fox RS, Torres TK, Badger TA, Katsanis E, Yang D, Sanford SD, Delivering a Group-Based Quality of Life Intervention to Young Adult Cancer Survivors via a Web Platform: Feasibility Trial. JMIR Cancer. 2024;10(1):e58014. doi:10.2196/58014 39631050 PMC11634045

[14] Irestorm E, Wakefield CE, Hetherington K, McGill BC, Evans HE, McDonald F, Recapturing Life: Virtual Peer-Based Psychological Support for Adolescent and Young Adult Cancer Survivors Delivered in the Community. J Adolesc Young Adult Oncol. 2025 Aug 11. doi:10.1177/21565333251369689 40794486

[15] Tutelman PR, Moran C, Beattie SM, Khu M, Howlett M, Scheidl J, Acceptability, feasibility and preliminary effects of an online group psychotherapy intervention for adolescents and young adults with cancer. Psychooncology. 2024;33(3):e6335. doi:10.1002/pon.6335 38526517

[16] Servais Z, Libert Y, Grégoire C, Lahaye M, Merckaert I. Psychological interventions in adolescents and young adults with cancer: a scoping review targeting protective factors of resilience. Curr Opin Oncol. 2025;37(4):312–23. doi:10.1097/CCO.0000000000001151 40464482

[17] Zhang A, Weaver A, Walling E, Zebrack B, Jackson Levin N, Stuchell B, Evaluating an engaging and coach-assisted online cognitive behavioral therapy for depression among adolescent and young adult cancer survivors: A pilot feasibility trial. J Psychosoc Oncol. 2023;41(1):20–42. doi:10.1080/07347332.2021.2011530 35040368 PMC10599691

[18] Collins LM, Nahum-Shani I, Guastaferro K, Strayhorn JC, Vanness DJ, Murphy SA. Intervention Optimization: A Paradigm Shift and Its Potential Implications for Clinical Psychology. Annu Rev Clin Psychol. 2024;20(1):21–47. doi:10.1146/annurev-clinpsy-080822-051119 38316143 PMC11245367

[19] Murphy KM, Glock R, Victorson D, Reddy M, Birken SA, Salsman JM. Co-Design of a Depression Self-Management Tool for Adolescent and Young Adult Cancer Survivors: User-Centered Design Approach. JMIR Form Res. 2025;9:e67175. doi:10.2196/67175 40126551 PMC11976180

[20] Murphy KM, Westcott G, Glock R, Coffey A, Augustinovic A, Yang D, User-centered design of a depression self-management tool for adolescent and young adult cancer survivors: Rapid qualitative analysis of individual interviews. JMIR Form Res. Under Review. 10.2196/77994 PMC13122137 41973497

[21] Ingram KM, Glock R, Williamson L, Coffey A, Anctil R, Augustinovic A, Evaluating the feasibility and acceptability of study procedures for a full factorial trial of ASCENT: protocol for a randomized pilot. Pilot Feasibility Stud. 2025;11(1):147. doi:10.1186/s40814-025-01728-z 41287126 PMC12642116

[22] McQuaid JR, Lin EH, Barber JP, Breland-Noble AM, Cuijpers P, Greenberg LS. Clinical practice guideline for the treatment of depression across three age cohorts. Wash DC Am Psychol Assoc. 2019.

[23] Cuijpers P, Karyotaki E, Harrer M, Stikkelbroek Y. Individual behavioral activation in the treatment of depression: A meta analysis. Psychother Res. 2023;33(7):886–97. doi:10.1080/10503307.2023.2197630 37068380

[24] Beck AT, Rush AJ, Shaw BF, Emery G, DeRubeis RJ, Hollon SD. Cognitive Therapy of Depression. Guilford Publications; 2024. 426 p.

[25] Segal Z, Williams M, Teasdale J. Mindfulness-based cognitive therapy for depression [Internet]. Guilford press; 2012 [cited 2024 Sep 6]. Available from: https://books.google.com/books?hl=en&lr=&id=1_NcsDZ17icC&oi=fnd&pg=PP1&dq=Segal+ZV,+Williams+M,+Teasdale+J.+Mindfulness-based+cognitive+therapy+for+depression.+Guilford+Publications%3B+2018.&ots=UD5hhBELI5&sig=_70UsLibTfyzYQRoToTBCm-D88s

[26] Hendriks T, Schotanus-Dijkstra M, Hassankhan A, De Jong J, Bohlmeijer E. The Efficacy of Multi-component Positive Psychology Interventions: A Systematic Review and Meta-analysis of Randomized Controlled Trials. J Happiness Stud. 2020;21(1):357–90. doi:10.1007/s10902-019-00082-1

[27] Epic. 2024.

[28] Harris PA, Taylor R, Thielke R, Payne J, Gonzalez N, Conde JG. Research electronic data capture (REDCap)–A metadata-driven methodology and workflow process for providing translational research informatics support. J Biomed Inform. 2009;42(2):377–81. doi:10.1016/j.jbi.2008.08.010 18929686 PMC2700030

[29] Harris PA, Taylor R, Minor BL, Elliott V, Fernandez M, O’Neal L, The REDCap consortium: Building an international community of software platform partners. J Biomed Inform. 2019;95:103208. doi:10.1016/j.jbi.2019.103208 31078660 PMC7254481

[30] Microsoft Teams [Internet]. Microsoft Corporation; 2024. Available from: https://www.microsoft.com/en-us/microsoft-teams

[31] Pilkonis PA, Choi SW, Reise SP, Stover AM, Riley WT, Cella D, Item Banks for Measuring Emotional Distress From the Patient-Reported Outcomes Measurement Information System (PROMIS^®^): Depression, Anxiety, and Anger. Assessment. 2011;18(3):263–83. doi:10.1177/1073191111411667 21697139 PMC3153635

[32] Watkins DC. Rapid and Rigorous Qualitative Data Analysis: The “RADaR” Technique for Applied Research. Int J Qual Methods. 2017;16(1):1609406917712131. doi:10.1177/1609406917712131

[33] Devine KA, Viola AS, Coups EJ, Wu YP. Digital Health Interventions for Adolescent and Young Adult Cancer Survivors. JCO Clin Cancer Inform. 2018;(2):1–15. doi:10.1200/CCI.17.00138 PMC6547376 30652583

[34] McCann L, McMillan KA, Pugh G. Digital Interventions to Support Adolescents and Young Adults With Cancer: Systematic Review. JMIR Cancer. 2019;5(2):e12071. doi:10.2196/12071 31368438 PMC6693302

[35] Zhang A, Zebrack B, Acquati C, Roth M, Levin NJ, Wang K, Technology-Assisted Psychosocial Interventions for Childhood, Adolescent, and Young Adult Cancer Survivors: A Systematic Review and Meta-Analysis. J Adolesc Young Adult Oncol. 2022;11(1):6–16. doi:10.1089/jayao.2021.0012 33960845 PMC8864427

[36] Kepper MM, Walsh-Bailey C, Zhao M, Parrish L, Miller ZM, Glasgow RE, Satisfaction and effectiveness of a digital health tool to improve health behavior counseling among adolescent and young adult cancer survivors: a randomized controlled pilot trial. BMC Digit Health. 2024;2(1):10. doi:10.1186/s44247-024-00064-1

[37] Corke L, Langelier DM, Gupta AA, Capozza S, Antonen E, Trepanier G, A Pilot Study to Evaluate the Feasibility and Acceptability of a Tailored Multicomponent Rehabilitation Program for Adolescent and Young Adult (AYA) Cancer Survivors. Cancers. 2025;17(7):1066. doi:10.3390/cancers17071066 40227632 PMC11988022

[38] Wurz A, Brunet J. Exploring the feasibility and acceptability of a mixed-methods pilot randomized controlled trial testing a 12-week physical activity intervention with adolescent and young adult cancer survivors. Pilot Feasibility Stud. 2019;5(1):154. doi:10.1186/s40814-019-0530-6 31890266 PMC6925485

[39] Miller I, Peake E, Strauss G, Vierra E, Koepsell X, Shalchi B, Self-Guided Digital Intervention for Depression in Adolescents: Feasibility and Preliminary Efficacy Study. JMIR Form Res. 2023;7(1):e43260. doi:10.2196/43260 37991839 PMC10701656

[40] Reading JM, Solk P, Starikovsky J, Hasanaj K, Wang SD, Siddique J, Optimization of a mHealth Physical Activity Promotion Intervention With Mindful Awareness for Young Adult Cancer Survivors: Design and Methods of Opt2Move Full Factorial Trial. Glob Adv Integr Med Health. 2024;13:27536130241265669. doi:10.1177/27536130241265669 PMC11325329 39149166

[41] Salsman JM, Murphy KM, Addington EL, Tooze JA, McLouth LE, Yang D, Optimization of a digital health intervention to enhance well-being among adolescent and young adult cancer survivors: Design and methods of the EMPOWER full factorial trial. Contemp Clin Trials. 2025;149:107783. 39710338 10.1016/j.cct.2024.107783 PMC11788047

[42] Harlan LC, Lynch CF, Keegan THM, Hamilton AS, Wu XC, Kato I, Recruitment and follow-up of adolescent and young adult cancer survivors: the AYA HOPE Study. J Cancer Surviv. 2011;5(3):305–14. doi:10.1007/s11764-011-0173-y 21274648 PMC3159756

[43] Freyer DR, Seibel NL. The Clinical Trials Gap for Adolescents and Young Adults with Cancer: Recent Progress and Conceptual Framework for Continued Research. Curr Pediatr Rep. 2015;3(2):137–45. doi:10.1007/s40124-015-0075-y 30613438 PMC6319956

[44] Vlooswijk C, van dePoll-Franse LV, Janssen SHM, Derksen E, Reuvers MJP, Bijlsma R, Recruiting Adolescent and Young Adult Cancer Survivors for Patient-Reported Outcome Research: Experiences and Sample Characteristics of the SURVAYA Study. Curr Oncol. 2022;29(8):5407–25. doi:10.3390/curroncol29080428 36005166 PMC9406992

[45] Aschbrenner KA, Kruse G, Gallo JJ, Plano Clark VL. Applying mixed methods to pilot feasibility studies to inform intervention trials. Pilot Feasibility Stud. 2022;8(1):217. doi:10.1186/s40814-022-01178-x 36163045 PMC9511762

[46] Creswell JW, Plano Clark VL. Designing and conducting mixed methods research. 2nd edition. Los Angeles London New Dehli Singapore Washington DC: Sage; 2011. 457 p.

[47] Miech EJ, Rattray NA, Flanagan ME, Damschroder L, Schmid AA, Damush TM. Inside help: An integrative review of champions in healthcare-related implementation. SAGE Open Med. 2018;6:2050312118773261. doi:10.1177/2050312118773261 PMC5960847 29796266

[48] Default [Internet]. [cited 2026 Jan 15]. COG Registry – Project:EveryChild. Available from: https://childrensoncologygroup.org/cog-registry-project-everychild

